# Stigmatization related COVID-19 and PTSD among Chinese graduates

**DOI:** 10.1186/s12888-022-04073-0

**Published:** 2022-06-29

**Authors:** Jingwen Gu, Juan Song, Jing Wang, Tuanjie Liu, Jingye Zhan, Wenjie Yan, Yanpu Jia, Lili Wu, Jing Xu, Weizhi Liu, Zhilei Shang

**Affiliations:** 1 Lab for Post-Traumatic Stress Disorder, Faculty of Psychology and Mental Health, Naval Medical University, #800 Xiangyin Road, Shanghai, 200433 China; 2 The Battalion 3 of Cadet Brigade, School of Basic Medicine, Naval Medical University, Shanghai, 200433 China; 3 Department of Medicine & Therapeutics, Faculty of Medicine, The Chinese University of Hong Kong, Hong Kong, 999077 China; 4 Department of Neurology, Wusong Central Hospital, Baoshan District, Shanghai, 200940 China; 5 Shanghai Key Laboratory of Mental Health and Psychological Crisis Intervention, School of Psychology and Cognitive Science, East China Normal University, Shanghai, 200062 China; 6 Psychological Counseling Center, Shanghai University, Shanghai, 200444 China

**Keywords:** PTSD, Stigmatization, COVID-19

## Abstract

**Background:**

Since COVID-19 broke out worldwide, it had caused extensive public health concerns and psychological distress, including PTSD and stigmatization towards recovered patients and people from high-risk areas. However, the association between PTSD, stigmatization and certain related factors have not been confirmed.

**Methods:**

Through cluster random sampling, 946 Chinese graduates were investigated from 5 universities in Shanghai at three months after China lifted its coronavirus lockdown. PTSD symptoms were evaluated with PCL-5. Demographic and disease-related characteristics including stigmatization, educational attainment and working position were collected to assess their association with PTSD.

**Results:**

12.4% graduates were reported significant PTSD symptoms in PCL-5 screening with a cut-off of 33. Graduates with a Master’s degree (*P* = 0.02) or working position like “looking for a job” and “planning to go abroad” (*P* = 0.038) showed severer stigmatization related to COVID-19. Stigmatization towards both patients recovering from COVID-19 and people from high-risk areas had significant association with PTSD symptoms. Multivariate linear regression analysis showed that stigmatization can explain 5% of variation of PCL-5 scores after controlling gender, age, educational attainments and working position.

**Conclusion:**

Graduates who were looking for jobs or preparing to go abroad showed more stigmatization related to COVID-19. There was a positive correlation between stigma against COVID-19 and PTSD symptoms. More attention should be paid to the mental health status of graduates who are preparing to go abroad or looking for jobs.

## Highlights

1. 12.4% Chinese graduates were positive for PTSD symptoms.

2. Graduates looking for a job or planning to go abroad showed severer stigmatization related to COVID-19.

3. People with severer stigmatization had more PTSD symptoms.

## Introduction

Globally, as of 16 May 2022, there have been 519,105,112 confirmed cases of Coronavirus Disease 2019 (COVID-19), including 6,266,324 deaths, which was reported by WHO [1]. The extremely high transmission rate of the coronavirus leaded to unprecedented prevalence of the pandemic worldwide, causing extensive public health concerns, psychological distress, as well as other mental health problems like insomnia and anxiety [2, 3]. Studies conducted in both high-income and low-income countries have all revealed an exacerbation of mental health problems during the pandemic among affected populations [4–6]. Previous researches showed that frontline healthcare worker had a higher risk of suffering from anxiety and depression [3]. Meanwhile, to college students, the outbreak of COVID-19 pandemic made their already intense mental health more difficult [7]. A study performed that college students had alarmingly higher stress and anxiety levels than the general population during the lockdown period [8]. In a word, the COVID-19 pandemic has posed great challenges to people’s mental health.

Post-traumatic stress disorder (PTSD) is a stress-related psychological problem that occurs in someone who experienced or witnessed a life-threatening traumatic event, which has contributed to a substantial burden on individuals and the society [9, 10]. In the literatures, PTSD correlated with self-stigma [11], current depression, previously negative experiences, as well as a history of psychopathology [12], with its prevalence rate at 13.10% ~ 18.8% in survivors of the traumatic events like genocide or earthquake [13–15]. The outbreak of COVID-19 were widely acknowledged as a severe traumatic event that imposed not only physical concerns but also psychological distress to the general public [16, 17]. Shortly after the outbreak of COVID-19, Liu et al. stated the prevalence of posttraumatic stress symptoms among residents of the initial outbreak place at the height of the pandemic to be 7% [18]. Moreover, those kept in quarantine were more likely to suffer from PTSD symptoms [19]. In accordance with former studies on acute and widescale stressors (e.g., natural disasters), studies have shown that COVID-19 was associated with a higher prevalence of PTSD [2, 20, 21].

Stigma is defined as an attribute that links a person to an undesirable stereotype, leading other people to reduce the bearer from a whole and usual person to a tainted, discounted one [22]. Stigmatization usually leads to discrimination and creates a sense of inequality among the people, convoying high levels of individual stress and significant health disparities [23]. Studies indicated that structural stigma (related to mental illness and sexual orientation) contributed to adverse mental health outcomes like dysregulated physiological stress responses [24]; similarly, discrimination also showed a significant positive relationship with depress, anxiety and post-traumatic stress disorder symptoms [25].

As studies [26, 27] suggested, psychological maladjustment to a severe pandemic can be associated with cultural factors such as individualism and collectivism. In line with other studies [28, 29], people of collectivistic orientations were found to associated with higher perceived vulnerability to infectious diseases than people with individualistic orientation, indicating more risk perception and sense of responsibility in collectivistic individuals when facing the COVID-19 pandemic [26]. Individuals who feel higher sociability with others, especially who feel a stronger sense of the integrity within one’s own family and support competitions of their in-groups against out-groups, may be more worried of being infected from others such as foreigners (out-group) or somebody unfamiliar. At the same time, they are more worried about infecting others such as family members, friends, and co-workers (in-group) through frequent contact. Therefore, we supposed that graduates (including bachelor’s, master’s and doctor’s degree) in a collectivist culture (like in China), especially who planned to go abroad to further their study or look for a job in a new place were more likely to be influenced by stigmatization. A study revealed that stigmatization related to COVID-19 was a consistent risk factor for predicting symptoms of depression; in addition, the same pattern for anxiety symptoms has also been observed [30, 31]. Also, it has been reported that the conditions of disrupted daily life and delays in academic activities were positively linked to the declining of students’ mental health condition [32], rising unhappiness, and conflict [33]. It can be thus predicted that for the group of students mentioned, the COVID-19 related stigmatization could negatively impact their mental health condition with the fear of being infected and stigmatized by others.

Although some researchers reported the roles of stigmatization related to COVID-19 as risk factors associated with mental health problems, there was no study identifying the association between COVID-19-related stigmatization and PTSD symptoms. The present study focused on the COVID-19 related stigmatization as well as its impact on mental health status among the graduates. It was believed that identifying the association of these risk factors with the mental health condition was conducive to the development of stigma targeted interventions towards individuals affected by COVID-19, as well as significant to the graduates’ decision-making for future during pandemic.

## Methods

### Participants and procedure

**Fig. 1:**
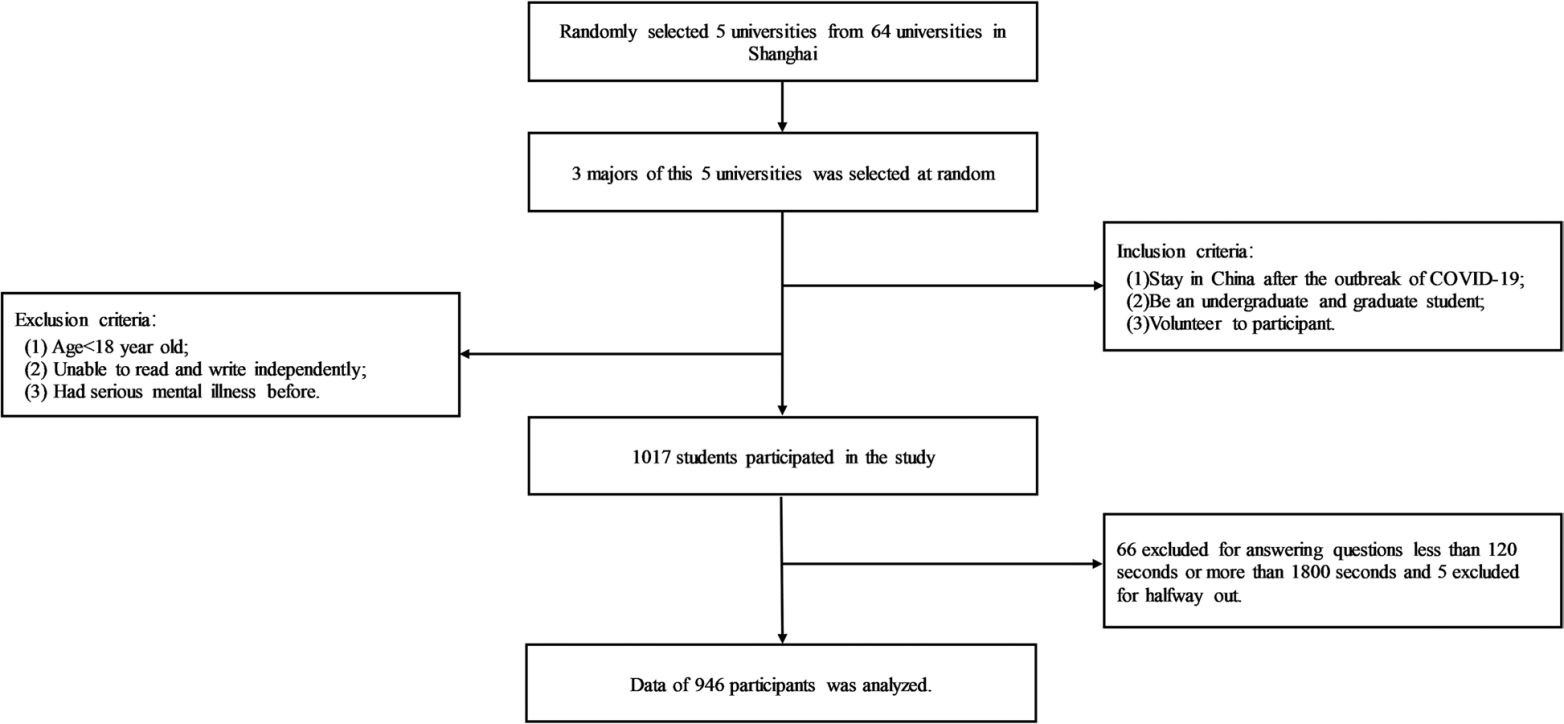
The sampling Flowchart

Through cluster random sampling (Fig. 1), we selected 5 universities at different levels out of 64 universities in Shanghai. To improve representativeness and reduce bias, both the first and the second batch of universities were included. At the same time, there were nor only natural sciences but also liberal arts students recruited to this research. The current study was designed to assess the relationship among potential stigmatization related to COVID-19, PTSD and possible influencing factors. The sample size was calculated with α set as 0.05, β as 0.2, and the overall prevalence of PTSD in college students estimated as 12%. A minimum of 704 graduates were required in this study. All data were collected between June and July, 2020, 3 months after China lifted its coronavirus lockdown. A mental health questionnaire was released through social networks Wechat. And one IP address could only fill in the questionnaire once. The inclusion criteria were as follows: (1) Staying in China after the outbreak of COVID-19; (2) Being an undergraduate and graduate student; (3) Volunteering to participate. And exclusion criteria were as follows: (1) Age < 18 years old; (2) Unable to read and write independently; (3) Had serious mental illness before. A total of 1,017 participants completed the questionnaire, and 946 of them entered the final analysis, with 66 excluded for answering questions less than 120 s or more than 1800s and 5 excluded for withdraw consent.

### Measures

#### Demographic characteristics

Information about gender (male and female), age (18–23, 24–29, ≥ 30), educational attainments (bachelor’s, master’s and doctor’s degree) were provided by participants. Meanwhile, working position (having found a job, continuing to further study, looking for a job, planning to go abroad) was also collected. The association between relevant risk factors and PTSD was analyzed based on different demographic characteristics.

#### PTSD symptoms

The symptoms of PTSD were measured by the Chinese Translated Version of PTSD Checklist for DSM-5 (PCL-5) [34]. PCL-5 is a 20-item self-report measure to assess the presence and severity of PTSD, corresponding with DSM-5 PTSD. Each item (the 20 items can be divided into 4 parts) is rated on a five-point Likert scale ranging from 0 (not at all) to 4 (extremely) according to how participants feel during the past month. All items are summed as a total severity score (range = 0–80) and a cut-off of 33 is used based on current psychometric work. A total score of 33 or higher suggests that the participant is PTSD positive. Meanwhile, if participant chooses the option of “moderately” in B items (at least once), C items (at least once), D items (at least twice) and E items (at least twice) respectively, the one also confirms to PTSD positivity according to the DSM-5 diagnostic rule. The Chinese Translated Version of PCL-5 is now widely used for early screening of PTSD symptoms [35, 36]. And the Cronbach α was 0.91 [35].

#### Stigmatization related to the COVID-19

Wuhan lifted its coronavirus lockdown in April, 2020, but neither was the COVID-19 completely under control nor effective medicine was found at that time. Previously, some researchers found that public attitudes towards the affected people was one of the elements when evaluated disease related stigma [37]. Therefore, we suspected that public panic of unwilling to be infected might lead to potential stigmatization during the pandemic. As there was no certain stigma scales attached to COVID-19, we set up 2 questions to assess the level of stigma, referring to commonly used stigma scales like Social Impact Scale (SIS) [38]. This 2 questions, about attitudes towards keeping in touch with patients recovering from COVID-19 and people from high-risk areas, were added to the questionnaire. Both questions were rated on a five-point Likert scale from 1 (not at all) to 5 (extremely) to assess stigmatization.

### Statistical analysis

Descriptive statistics were conducted with categorical variables presented as frequencies and percentage, and continuous variables presented as mean and standard deviation (SD). T-test was performed to compare the stigmatization related to COVID-19 and PCL-5 scores in different gender. And one-way ANOVA was run to test the differences in the stigmatization and PCL-5 scores between groups categorized according to age, educational attainments and working position, with LSD used for post hoc test. Meanwhile, analysis of covariance (ANCOVA) was used to explore the interaction between gender (and working position) and stigmatization on PTSD. Then, Pearson correlation were conducted to assess association between stigmatization related to COVID-19 and PTSD. Finally, multivariate linear regression model was used to examine the effect of stigmatizations on PCL-5 scores with all demographic characteristics controlled. Categorical variables with three or more groups were firstly transformed to dummy variables before entered into the model. Analyses were performed using IBM Statistical Package for Social Sciences version 26.0, and *p* value < 0.05 was considered as statistically significant.

## Result

### Sample description

Among 946 students included in the data analysis, 403 (42.6%) of them were male and 543 (57.4%) were female. 448 (47.4%) had found a job, 225 (23.8%) decided to further their study, 237 (25.1%) was looking for a job and 36 (3.8%) planned to go abroad. Besides, 743 (78.5%) graduates were with bachelor’s degree, 197 (20.8%) with master’s degree and 6 (0.6%) with doctor’s degree. The mean scores of participants’ stigmatization against patients recovering from COVID-19 was 1.78 (SD = 1.01) and 1.88 (SD = 1.01) in which against people from high-risk areas. Moreover, 117 (12.4%) graduates were positive in PCL-5 screening which indicated a significant PTSD symptoms, using a cut-off of 33. Item rated as 2 = “Moderately” or higher as a symptom endorsed, and 443 (46.8%) participants reported at least 1 Criterion B symptom, 333 (35.2%)with at least 1 Criterion C symptom, 323 (34.1%) with at least 2 Criterion D symptom, and 239 (25.3%) with at least 2 Criterion E symptom. Totally, there are 132 (14%) graduates showing significant PTSD symptom following the DSM-5 diagnostic rule (Table 1).

**Table 1:** Demographic characteristics and stigma-related characteristics of 946 participants

	**N/MEAN**	**%/SD**
**Gender**		
Male	403	42.6
Female	543	57.4
**Age**	23.28	2.05
18–23	647	68.4
24–29	285	30.1
≥ 30	14	1.5
**Educational attainment**		
Bachelor’s degree	743	78.5
Master’s degree	197	20.8
Doctor’s degree	6	0.6
**Working position**		
Have found a job	448	47.4
Continue further study	225	23.8
Look for a job	237	25.1
Go abroad or plan to go abroad	36	3.8
**Stigmatization against patients recovering from COVID-19**	1.78	1.01
**Stigmatization against people from high-risk areas**	1.88	1.01
**PCL-5 Scores**	17.39	13.73
< 33	829	87.6
≥ 33	117	12.4
Scores of B items	4.75	3.78
Scores of C items	2.17	2.13
Scores of D items	6.08	5.16
Scores of E items	4.39	4.38
**PTSD Positive**	132	14
> 1 Criterion B item	443	46.8
> 1 Criterion C item	333	35.2
> 2 Criterion D items	323	34.1
> 2 Criterion E items	239	25.3

*Note*: PCL-5:the PTSD Checklist for DSM-5

### Association among demographic characteristics, stigmatization and PTSD

Next, the association between demographic characteristics and stigmatization against patients recovering from COVID-19 was explored, the result showed significant discrepancies in educational attainments (F = 3.915, *P* = 0.02). Graduates with a master’s degree reported higher scores of stigmatization than those with bachelor’s (*P* = 0.010). The differences among various working position were also statistically significant (F = 2.825, *P* = 0.038). Graduates who planned to go abroad scored higher, compared with those willing to further study. There were no significant differences of stigmatization scores against people from high-risk area in groups of different gender, age and working position.

In term of PTSD symptoms, compared with male, female were much more likely to score higher in PCL-5 (t = -2.389, *P* = 0.017). Significant relationship was also found between working position and PCL-5 scores (F = 13.465, *P* < 0.001). The scores of PCL-5 screening were higher in graduates looking for a job (*P* < 0.001) or planning to go abroad (*P* = 0.002) than graduates had found a job. Meanwhile, compared with those who continued to further study, there was a higher risk to have PTSD in graduates who were looking for a job at that time (*P* < 0.001) and those planned to go abroad (*P* = 0.021). There were no significant association between PTSD and factors like age and educational attainment (Table 2).

**Table 2:** Demographic characteristics and stigma-related characteristics associated with stigmatization and PCL-5 scores

	**Stigmatization against patients recovering from COVID-19**		**F/t**	**p**	**Stigmatization against people from high-risk areas**		**F/t**	**p**	**PCL-5 Scores**		**F/t**	**p**
	**MEAN**	**SD**			**MEAN**	**SD**			**MEAN**	**SD**		
**Gender**			-0.333	0.739			-1.615	0.107			-2.389	0.017*
Male	1.76	1.06			1.82	0.99			16.16	13.16		
Female	1.79	0.97			1.93	1.03			18.31	14.08		
**Age**			1.714	0.181			1.795	0.167			0.218	0.804
18–23	1.74	1.01			1.84	1.00			17.59	14.11		
24–29	1.87	1.01			1.98	1.05			16.98	12.99		
≥ 30	1.86	0.77			1.86	0.77			16.57	11.13		
**Educational attainment**			3.915	0.02*			5.175	0.006**				
Bachelor’s degree	1.74	1.00			1.83	0.99			17.48	14.08	0.249	0.779
Master’s degree	1.94	1.03			2.07	1.10			17.15	12.35		
Doctor’s degree	1.33	0.52			1.33	0.52			13.83	14.36		
**Working position**			2.825	0.038*			0.969	0.407			13.465	< 0.001***
Have found a job	1.84	1.04			1.92	1.04			15.16	12.27		
Continue further study	1.65	0.91			1.79	0.96			16.67	11.37		
Look for a job	1.74	1.00			1.88	1.01			21.56	16.19		
Go abroad or plan to go abroad	2.06	1.15			1.94	1.07			22.28	19.11		

*Note*: ^*^p-value < 0.05; ^**^p-value < 0.01; ^***^p-value < 0.001

### Correlations between COVID-19 related stigmatization and PTSD

Correlations between COVID-19 related stigmatization and PCL-5 scores were presented in Table 3. Stigmatization and PTSD was significantly correlated (*p* < 0.01). Stigmatization against patients recovering from COVID-19 had significant positive correlation with the scores of PCL-5 and each item. Similar result was found in the stigmatization against people from high-risk areas.

**Table 3:** Corelations among stigmatizations, PCL-5 scores and scores of certain items

	**Stigmatization against patients recovering from COVID-19**	**Stigmatization against people from high-risk areas**	**PCL-5 Scores**	**Scores of B items**	**Scores of C items**	**Scores of D items**	**Scores of E items**
Stigmatization against patients recovering from COVID-19	1						
Stigmatization against people from high-risk areas	.729^a^	1					
PCL-5 Scores	.201^a^	.213^a^	1				
Scores of B items	.156^a^	.162^a^	.892^a^	1			
Scores of C items	.109^a^	.156^a^	.722^a^	.636^a^	1		
Scores of D items	.193^a^	.193^a^	.934^a^	.747^a^	.590^a^		
Scores of E items	.216^a^	.224^a^	.913^a^	.742^a^	.531^a^	.817^a^	1

*Note*: ^a^Corelation is significant at the 0.01 level

### Regression model

Multivariate linear regression analysis was performed to explore the effect of stigmatization related to COVID-19 on PTSD symptoms after controlling gender, age, educational attainments, and working position. The result was presented in Table 4. It showed significant relationship between stigmatization related to COVID-19 and PCL-5 scores (B = 1.46, t = 2.350, *P* = 0.019 for stigmatization against patients recovering from COVID-19; B = 1.88, t = 3.041, *P* = 0.002 for stigmatization against people from high-risk areas). Stigmatization can explain 5% of variation of PCL-5 scores after controlling gender, age, educational attainments and working position. Additionally, certain working position was positively associated with PCL-5 scores (looking for a job vs. having found a job: B = 6.57, t = 6.188, *P* < 0.001; going abroad vs. having found a job: B = 6.32, t = 2.754, *P* = 0.006).

**Table 4:** Regression model examing the effect of demographic characteristics and stigmatizations on PTSD positive result

	**B**	**SD**	**β**	**t**	**p**
**Gender**					
Female vs. Male	1.70	0.90	0.06	1.893	0.059
**Age**					
24–29 vs.18–23	-0.47	1.32	-0.02	-0.359	0.720
≥ 30 vs.18–23	2.10	4.46	0.02	0.471	0.637
**Educational attainment**					
Master’s degree vs.Bachelor’s degree	-1.51	1.53	-0.05	-0.992	0.322
Doctor’s degree vs.Bachelor’s degree	-2.09	6.54	-0.01	-0.320	0.749
**Working position**					
Continue further study vs.Have found a job	1.75	1.11	0.05	1.588	0.113
Look for a job vs.Have found a job	6.57	1.06	0.21	6.188	< 0.001^***^
Go abroad or plan to go abroad vs.Have found a job	6.32	2.30	0.09	2.754	0.006^**^
**Stigmatization against patients recovering from COVID-19**	1.46	0.62	0.11	2.350	0.019^*^
**Stigmatization against people from high-risk areas**	1.88	0.62	0.14	3.041	0.002^**^

*Note*: ^*^p-value < 0.05; ^**^p-value < 0.01; ^***^p-value < 0.001

### Interaction between demographic characteristics and stigmatization on PTSD

**Fig. 2:**
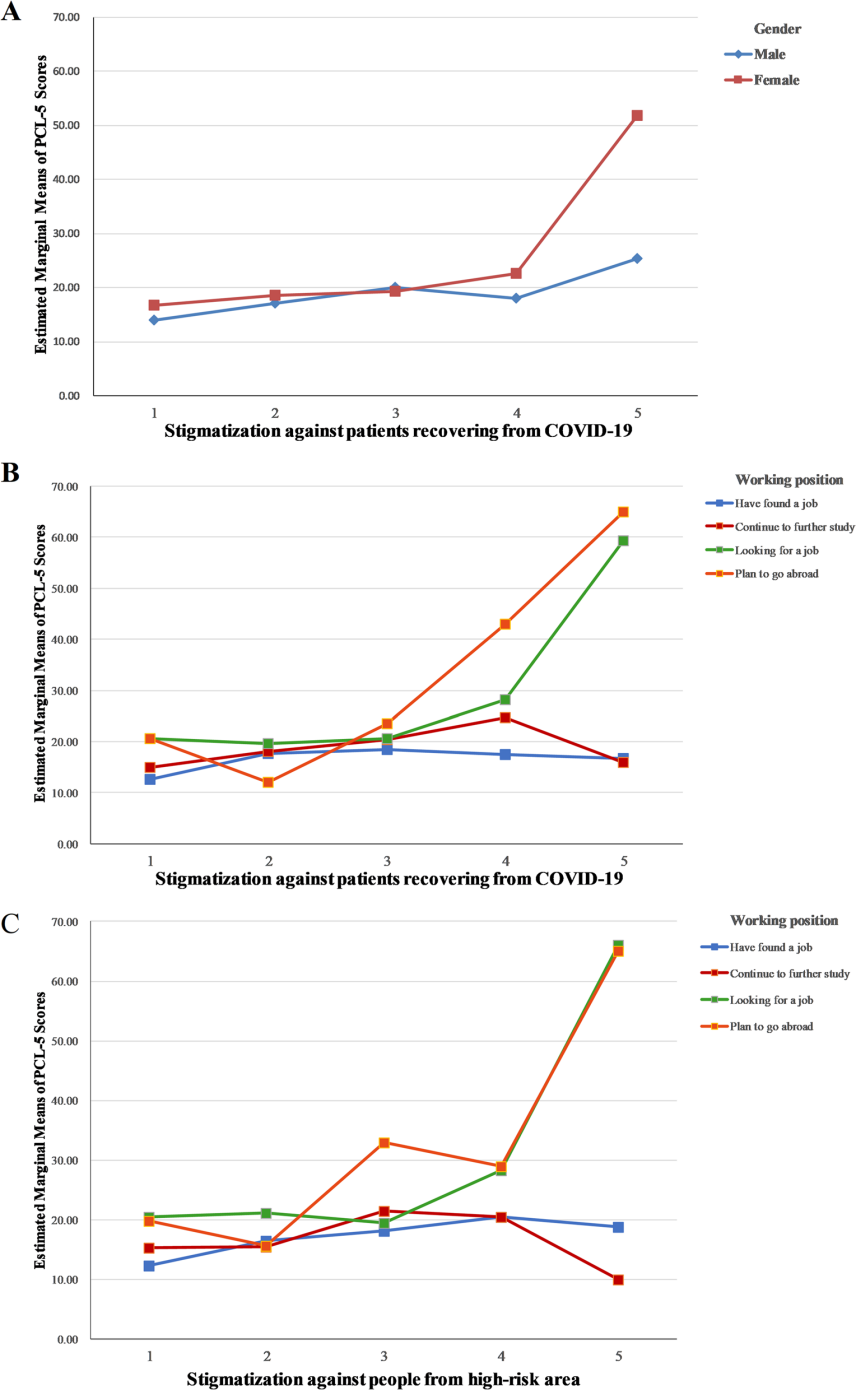
Interaction of socidemographic characteristics and stigmatization on PCL-5 scores

ANCOVA analysis suggested that the interaction of gender, stigmatization and PTSD was significant (F = 4.35, *P* = 0.002). Compared to male, female who scored higher in stigmatization against patients recovering from COVID-19 were more likely to obtain a higher score in PCL-5 screening (Fig. 2A). Similar result was observed when considering about the interaction between working position and stigmatization on PTSD (Fig. 2B, 2C). Among graduates looking for a job or planning to go abroad, when they showed discrimination towards patients recovering from COVID-19 or people from high-risk area, they were in higher risk of having PTSD, in comparison to those who have found a job or continued to further study (respectively, F = 4.898, *P* < 0.001; F = 4.961, *P* < 0.001).

## Discussion

The present study aimed to explore the association between stigmatization related to the COVID-19 (as well as other relevant risk factors) and PTSD among Chinese graduates during the pandemic. PCL-5 screening indicated 12.4% graduates presented significant PTSD symptoms (cut-off = 33). Stigmatization towards both patients recovering from COVID-19 and people from high-risk areas had significant association with PTSD symptoms. The effect of stigmatization against COVID-19 on PTSD symptoms were still significant after controlling gender, age, educational attainments and working position. Graduates who were looking for a job or planning to go abroad showed severer stigmatization against COVID-19 and more PTSD symptoms.

When facing the COVID-19 pandemic, people of collectivistic orientations (like in China) had higher perceived vulnerability to infectious diseases compared with those of individualistic orientation. Higher risk perception and sense of responsibility may lead to their overwhelming worry of being infected [26]. This was in accordance with most research results that public health emergencies can have many psychological impacts on the general public [38–42], especially college students by causing anxiety, fear, and worry among others [32].

Correlation between stigmatization related to COVID-19 and PTSD symptoms was found to be significant from our result, which had also been proved in other studies [11, 39, 43, 44]. Social stigma and self-stigma could explain the reason for this result. With the social media use, graduates may be exposed to some radical news or partial online speech about COVID-19, which have proved to be associated with sleep deprivation, emotional distress and anxiety [45]. Heightened fear of being infected will also cause social stigma, including social rejection, social isolation and financial insecurity [38]. During the pandemic, the worry of being infected by “RE-positive” patients (i.e., patients who met criteria for hospital discharge testing positive for COVID-19 again) was not proportional to the probability of the threat [46]; that is, though cured patients testing positive again were small probability events, people’s perceived threat of infection and the following worry were likely to be at higher levels. Meanwhile, people of collectivistic orientations have proved to have a stronger sense of integrity with their own family and social groups. In this social background, they will be more worried about infecting the closest or experiencing social isolation, which may lead to potential self-stigma [26]. Also, a research found that the association among mental problems, COVID-19 related self-stigma and their quality of life [39, 42]. Overall, it has been proved that stigmatization by the individuals and communities, in the context of trauma itself would act as a factor in the continuity of the PTSD; this effect could be beyond the severity of trauma [44]. The perceived stigmatization, in this case, could aggravate PTSD psychopathology and prevent symptoms from improving [44].

As our results showed, gender and working position, two demographic characteristics in the present study, were significantly associated with PCL-5 scores. Females were statistically associated with higher PCL-5 scores compared to males, which was consistent with previous studies [3, 18, 47]. College students bore a disproportionate burden of mental health problems worldwide, with females having higher anxiety and depression levels than males [7]. The former studies also claimed that females tended to be more vulnerable to PTSD with less power in decision making during the pandemic, which probably furthered negative cognitive changes in this situation [18], as well as largely unmet needs [48]. However, gender was no longer a significant risk factor of PTSD after considering the effect of stigmatization in the regression model, which can be interpreted as the weakening effect of the gender due to the difference in stigmatization’s impact on male and female. As shown in the further analysis, the interaction of gender, stigmatization towards patients recovering from COVID-19 and PTSD was significant, that was a closer link between stigmatization and PTSD was found in female.

As suggested in the present study, differences in both stigmatization against recovered patients and PCL-5 scores among different working position were statistically significant. To those who decided to further their study, as the government took measures to control the outbreak, including extending the national holidays, postponing classes at universities or using distant/remote learning methods [49, 50], which inevitably disrupted routine study life and resulted in anxiety among students [32]. For graduates who were looking for a job at that time and planning to go abroad, they had greater probability to come into contact with unfamiliar people and foreigners. This raised their risk of infection and also they might receive stigmatization from people in the new environment. Besides, as graduates in a collectivist culture, they were also afraid of infecting family members or friends, leading huge stress and anxiety [7, 26, 35]. Meanwhile, worrying about stability of income also turned out to be a significant factor in college students’ experienced anxiety [32]; therefore, as we predicted, graduates with such working position had a higher risk to develop PTSD with higher PCL-5 scores than students with a job, which was also proved in the present study. Interestingly, graduates who continued to further study scored lower in stigmatization than those who went abroad or planned to go abroad. It made sense that with more understanding of the relatively extensive prevalence of COVID-19 in major countries and regions out of China, graduates with the plan of going abroad tended to display higher discrimination against recovered patients with the worry of being infected by “RE-positive” ones among them, which was common in PTSD community as mentioned above [1].

Further analysis showed the significant interaction of working position, stigmatization and PTSD. Participants looking for a job and planning to go abroad were at greater risk of having PTSD when displaying discrimination related to COVID-19 compared with those with a stable job. With millions of people losing access to employment [51] and the US national unemployment rate rivaling only by that of the Great Depression, the pandemic has exerted a negative impact on employment stability [52]; in previous studies, those who struggled to find and maintained employment without relief of new work suffered a higher risk of psychological harm [53], while job search process itself can also result in decreased psychological well-being [54]. Therefore, it was natural that with more stigmatization against COVID-19, this negative psychological impact will be amplified in those vulnerable job hunters; also, for those who planned to go abroad where relatively higher infection rate among the public, greater discrimination related to COVID-19 will lead to more worry of being infected as well as higher risk of having PTSD.

For a significant number of young people, the COVID-19 pandemic represented a seismic psychological event, but also led to posttraumatic growth (PTG) [3, 55, 56]. PTG has been proved to be related to factors like social support, good mental health (i.e., lower level of depression and anxiety) and higher life quality, which should gain special attentions. Government and society should offer more objective supports and services to improve young people’s mental condition [56]. Certain strategies, such as mindfulness interventions and prioritizing positivity, can be instituted quickly to reduce the adverse psychosocial impact caused by COVID-19 [3, 55].

### Limitations

For this study, there were a few limitations worth our attention. First, this was a cross-sectional survey. Therefore, some factors that may affect the results, such as the mental health level before the pandemic, were not collected. Secondly, the conclusion related to PTSD drawn from the selected group may need further verification. Besides, our study used PCL-5 scale through PTSD screening, which was not a clinical diagnosing method. The accuracy for PTSD diagnosis may thus be limited due to lack of professional assessment in medical environment. Moreover, as the use of self -report record, the results undeniably had the limitation of recall bias and interview bias.

## Conclusion

The present study found that 12.4% Chinese graduates were positive for PTSD. Stigmatization against both patients recovering from COVID-19 and people from high-risk areas was found to be correlated with higher PCL-5 scores. Graduates who were looking for jobs or preparing to go abroad showed more stigmatization related to COVID-19 and higher PCL-5 scores.

As our result shows, gender and working position, two demographic characteristics in the present study, were significantly associated with PCL-5 scores. However, gender was no longer a significant risk factor of PTSD after considering the effect of stigmatization in the regression model, while working position among graduating students, (including looking for a job vs. having found a job, going abroad or planning to go abroad vs. having found a job) remained significant. This can be explained by the significant interaction of gender, stigmatization and PTSD, i.g. female with higher stigmatization against recovered patients tend to score higher in PCL-5 screening. As for the interaction between working position and stigmatization on PTSD, graduates looking for a job or planning to go abroad displayed higher risk of having PTSD, when they showed discrimination towards recovered patients of COVID-19 or people from high-risk area, in comparison to those with a stable job or continued to further their study.

Therefore, more attention should be paid to the mental health status of graduates who are preparing to go abroad or looking for jobs. And stigmatization among the public should be paid attention and stigmatization-oriented interventions towards certain groups are needed to foster post-traumatic growth among young people.

## Data Availability

The datasets of current study are not publicly available due to the relatedness of the recruited data to Wusong Central Hospital Zhongshan Hospital Fudan University, but are available from the corresponding author on reasonable request.

## Abbreviations

COVID-19: Coronavirus Disease 2019
PTSD: Post-traumatic stress disorder
PCL-5: PTSD Checklist for DSM-5
SIS: Social Impact Scale
PTG: Posttraumatic Growth
